# Comparison of bone age assessment using manual Greulich and Pyle method versus automated BoneXpert method in South African children

**DOI:** 10.4102/sajr.v29i1.3033

**Published:** 2025-04-11

**Authors:** Radhiya Minty, Nasreen Mahomed, Nicole van Wyk, Gopolang Mndebele, Zarina Lockhat, Ashesh Ranchod

**Affiliations:** 1 Department of Radiology, Faculty of Health Sciences, University of the Witwatersrand, Johannesburg, South Africa; 2 Department of Paediatrics and Child Health, Faculty of Health Sciences, University of the Witwatersrand, Johannesburg, South Africa; 3 Department of Radiology, Faculty of Health Sciences, University of Pretoria, Pretoria, South Africa

**Keywords:** bone age assessment, Greulich and Pyle, BoneXpert, artificial intelligence, skeletal maturity

## Abstract

**Background:**

The Greulich and Pyle (GP) method is the most commonly used manual bone age assessment method but it is associated with interrater variability. The BoneXpert method is fully automated, eliminates interrater variability and has been validated for use in various populations.

**Objectives:**

To compare the manual GP method with the automated BoneXpert method in performing bone age assessment of children with various paediatric endocrinology diagnoses.

**Method:**

Three manual readers performed manual bone age assessment, and BoneXpert software performed automated bone age assessment on 260 left hand-wrist radiographs. Images where the average of three manual readers (Manual BA) deviated from BoneXpert BA by > 1.5 years, were re-read by an external reader, producing a Reference BA. Manual BA was compared to Carpal BA that was produced by the software. A composite bone age (Comp BA) for the software was defined to estimate the weighting on carpal and tubular bones to achieve the best agreement with Manual BA.

**Results:**

The interclass correlation (ICC) between each manual reader was > 0.9, indicating a high positive correlation. The ICC between Manual BA and BoneXpert BA was 0.982. The Comp BA for BoneXpert that would achieve the best fit with Manual BA, places a 50% weighting on Carpal BA and 50% weighting on Tubular BA.

**Conclusion:**

The BoneXpert method is efficient, well-validated and shows a positive correlation with the manual GP method. An estimated weightage of 50% to carpal bones and 50% to tubular bones resulted in an automated Comp BA with the best agreement with Manual BA.

**Contribution:**

This original research article compares manual and automated bone age assessment methods to evaluate the use of artificial intelligence tools in the South African context.

## Introduction

Skeletal maturity is represented by the degree of ossification in bone and can be measured using bone age assessment.^1^ Every bone progresses through a relatively consistent sequence of changes that can be observed radiologically. The most widely used method for assessing skeletal maturity uses hand-wrist radiographs to determine bone age, which is correlated with the patient’s chronological age.^2^

Bone age assessment is a routine task for general radiologists and paediatric radiologists. It is requested by paediatricians and paediatric endocrinologists as part of the diagnostic work-up for patients with diseases that cause tall or short stature. Serial measurements can be performed to monitor response to treatment for these diseases.^3^ Bone age determination can also be used to estimate patients’ chronological ages with absent or unknown birth records. There are various radiological methods available to calculate bone age.

### Manual methods of bone age assessment

The most commonly used manual method is the Greulich and Pyle (GP) Atlas, first published in 1950, displaying images of left-hand and wrist radiographs for males and females of different ages.^4^ The patient’s hand and wrist x-ray is compared to the images in the atlas to assign a bone age. By analysing the carpals, metacarpals and phalanges, the most similar reference image in the atlas becomes the patient’s bone age.

The Tanner–Whitehouse (TW) method is the second most commonly used method.^5^ It involves assessing 20 individual bones in the hand and wrist and assigning a numerical score for each bone’s stage of development. The sum of these scores correlates to a particular bone age. The TW method is more accurate than the GP method, but more time-consuming.^1^

The recently developed Gilsanz and Ratib Atlas is a digital atlas featuring artificial images that are of better quality than those in the GP Atlas.^6^ The BonAge ultrasound device involves sonographic examination of the distal radius and ulna epiphysis to determine skeletal age using gender and ethnicity-based algorithms.^7^ This method is still in its initial stages and requires further evaluation in more extensive sample studies.^6^ Bone age can also be determined by visualisation of dental maturity.^6^ Various atlases can be used to compare mineralisation in teeth to reference orthopantomography.^6^

The clavicle is the last ossification centre to fuse at approximately 22 years of age. Bone age for individuals 18–22 years old has been calculated using visualisation of the clavicle on conventional radiographs, CT and MRI.^8^ Other less commonly used methods of bone age determination are visualisation of the iliac crest apophysis (Risser sign) and femoral epiphysis.^6^

For decades, bone age assessment has been conducted manually using the GP or TW methods. Both methods require considerable time and are subject to interrater variability.^9^ Furthermore, the GP skeletal maturity standards are based on a single ethnic group of children and may not apply to children of diverse ethnicities and different socioeconomic backgrounds.^10^

### Automated methods for bone age assessment

Artificial intelligence (AI) can be defined as software that automates a manual task.^11^ There has been tremendous progress in applying AI principles to diagnostic radiology. Tools in AI can be divided into three categories, namely, AI-assist (assisting the radiologist), AI-replace (replacing the radiologist) and AI-extend (AI that performs tasks beyond what is humanly capable by the radiologist).^11^

To encourage the development of radiology AI tools, the Radiology Society of North America (RSNA) conducted the RSNA Pediatric Bone Age Machine Learning Challenge in 2017.^12^ Participants who registered for the challenge were provided with a data set of 14 236 hand radiographs from Stanford University and the University of Colorado.^12^ BoneXpert software ranked fourth among the top five best submissions.^12^

The BoneXpert software was first introduced in April 2008 by the company Visiana, Horsholm, Denmark.^13^ As of April 2022, the software is being utilised by more than 200 radiology departments, predominantly in Europe.^14^ BoneXpert was initially intended to be an AI-replace tool.^14^ According to a survey conducted in 2020–2021 across 149 radiology departments within the European Union, most radiologists use BoneXpert as an AI-assist tool.^14^ An additional AI-assist (and potentially AI-extend) function is the BoneXpert Adult height prediction method. This replaces the manual Bayley and Pinneau method.

## Research methods and design

This study was designed as a retrospective, multicentre, comparative study. All left hand and wrist radiographs performed for bone age assessment between 01 June 2020 and 31 May 2023 were included in the study. The relevant patients were identified using records from the paediatric endocrinology clinics and radiology departments of Chris Hani Baragwanath Academic Hospital (CHBAH), Charlotte Maxeke Johannesburg Academic Hospital (CMJAH) and Rahima Moosa Mother and Child Hospital (RMMCH). Permission was granted by the heads of the radiology departments and hospital Chief Executive Officers (CEOs) at all three hospitals.

Radiographs were acquired on Shimadzu UD150V-40 X-ray units at both CHBAH and CMJAH, and a Philips DigitalDiagnost X-ray unit at RMMCH. All radiographs were digital. A good quality hand radiograph for bone age assessment should include the distal forearm and fingertips. The hand should be positioned with the palm down flat, fingers flat and separated. No flexion of any joints or rotation of the hand should be observed.

All radiographs were anonymised, assigned a numerical identifier and stored in JPEG format. For clinical use, BoneXpert’s intended image format is DICOM image files. However, for research purposes, additional supported image file formats supported include JPEG, PNG and BMP files. In this study, the images were analysed in ‘unknown resolution’ and only the gender was entered for each uploaded image. If no resolution is provided, BoneXpert assigns an inferred resolution, which is derived by comparing the detected hand size in the image with expected hand dimensions that are built into its model.

### Manual bone age assessment

Manual bone age assessment of all the radiographs in the data set was performed independently by three manual readers using the GP atlas : a general radiologist, Reader 1, a paediatric endocrinologist, Reader 2 and a paediatric radiologist, Reader 3. For cases where the bone age was assessed to lie between two standards, an average of the two values was assigned as the bone age. The manual readers also observed other incidental findings on the radiographs.

### Automated bone age assessment

**FIGURE 1:**
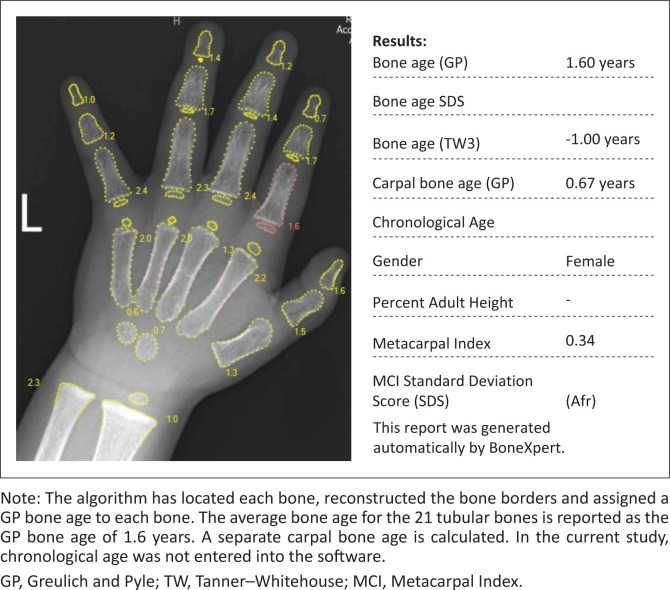
Dorso-palmar left hand and wrist radiograph of a 2-year 1-month-old girl, assessed by BoneXpert.

Automated bone age assessment was performed using BoneXpert Standalone Version 3.2.2 (Visiana, Holte, Denmark, www.boneXpert.com). BoneXpert analysed 28 bones – 19 short bones, radius, ulna and seven carpal bones. The analysis produces a GP bone age, TW bone age, carpal bone age and bone health index. The GP bone age produced by the software is based on the 21 tubular bones (BoneXpert BA). The carpal bone age based on seven carpal bones is produced as a separate reading (Carpal BA) (Figure 1). Carpal bone age is computed by the software up to a carpal bone age of 11 years in boys and 9 years in girls.

The image processing is divided into three layers:^15^ Layer A locates each bone, outlines the bone borders and validates the bone borders. At this stage, bones with abnormal morphology may be rejected; Layer B calculates the bone age for each bone based on shape, bone density and texture, formulating an average bone age. Any bone with a bone age that differs more than 2.4 years from the average bone age, is rejected; Layer C converts the intrinsic bone age into GP or TW bone ages^15^

Images with a discrepancy of >1.5 years between the average of the manual bone age readings (Manual BA) and automated readings (BoneXpert BA) were re-read by an external reader, producing a reference bone age reading (Reference BA). The differences between Reference BA and Manual BA, Reference BA and BoneXpert BA, and Manual BA and Carpal BA were calculated.

Manual readers report one bone age from the hand and wrist radiograph and there is no standard for the weighting placed on the carpals and tubular bones during this assessment. Therefore, nine different composite bone ages (Comp BA) for BoneXpert were calculated, with 10%, 20%, 30% etc. up to 90% weighting on the carpals. The following formulae were used to calculate the composite bone ages, where factors (*f*-values) of 0.1 up to 0.9 were used to compute different composite bone ages: Comp BA = (*f*) Carpal BA + (1–*f*) Tubular BA and Comp BA = Tubular BA (in cases where Carpal BA was not computed). All nine Comp BA (*f* = 0.1 up to *f* = 0.9) were compared and the best fit with Manual BA was determined (Online Appendix 1).

### Data analysis and statistics

Results were recorded in an Excel spreadsheet. Statistical analysis was performed using IBM SPSS statistics version 29. Patients’ demographic information and clinical indications were reported in counts and percentages (for categorical variables) and in means with standard deviation (for continuous variables). The level of agreement between each manual reader was assessed using the intraclass correlation. The average manual bone age of the three readers (Manual BA) was compared to the automated bone age readings (BoneXpert BA). Manual BA was then compared to Comp BA (for different *f* values *f* = 0.1 up to *f* = 0.9). This was performed through Bland–Altman plots to visualise the mean differences in the Manual BA versus BoneXpert BA and Manual BA versus Comp BA and scatterplots produced the coefficient of determination (*R*^2^). The root mean square error (RMSE) was also computed to show the average differences between Manual BA and Comp BA. For all the statistical analyses *p* < 0.05 indicated statistical significance.

### Ethical considerations

The University of Witwatersrand’s Human Research Ethics Committee granted ethical clearance to conduct this study (clearance certificate no. M230930).

## Results

A total of 260 left-hand and wrist radiographs from 152 males and 108 females were collected. The chronological age range was 0.2–18.9 years for males (mean age of 9 years) and 0.4–18.3 years for females (mean age of 9.2 years). The study population consisted of South African children of different ethnicities. The clinical indications for performing bone age assessment are listed in Table 1.

**TABLE 1:** The clinical indications for bone age assessment.

Indication	*n*	%
**Short stature**	**116**	**44.6**
Idiopathic short stature	33	12.7
Growth Hormone Deficiency	63	24.2
Short stature secondary to chronic illness	13	5.0
Hypothyroidism	7	2.7
**Tall stature**	**44**	**16.9**
Precocious puberty	32	12.3
Increased body mass index	11	4.2
Hyperthyroidism	2	0.8
**Growth monitoring**	**63**	**24.2**
Syndromes	21	8.1
Hypogonadism	19	7.3
Congenital adrenal hyperplasia	16	6.2
Addison’s disease	4	1.5
Turner syndrome	3	1.2
**Brain tumours**	**28**	**10.8**
Craniopharyngioma	14	5.4
Pituitary microadenoma	7	2.7
Hypothalamic harmatoma	2	0.8
Pituitary cyst	1	0.4
Suprasellar cyst	1	0.4
Suprasella PNET	1	0.4
Germocystic astrocytoma	1	0.4
Medulloblastoma	1	0.4
**Other**	**9**	**3.5**
Skeletal dysplasia	3	1.2
Intellectual disability	3	1.2
Dysmorphism	2	0.8
Rheumatological disorders	1	0.4

**Total**	**260**	**100.0**

**FIGURE 2:**
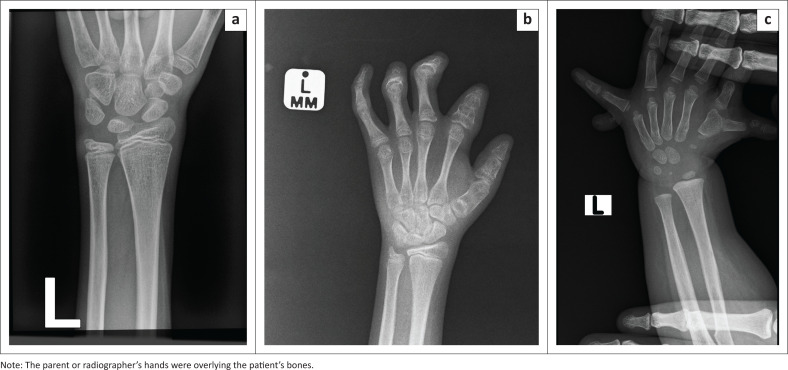
Rejected images: (a) Rejected because of insufficient bones; in this case only the wrist was imaged (b) Rejected because of abnormal pose of the bones, there were flexion deformities of the distal interphalangeal joints (c) Rejected because of foreign object superimposed on the bones.

BoneXpert analysed 254 images and rejected six images, resulting in an efficiency of 97.7%. The reasons for the six rejected images were: insufficient bones (*n* = 2), bone in an incorrect pose (*n* = 2) and foreign object superimposed on the bone image (*n* = 2). (Figure 2).

Manual readers noted other radiological findings in 22 of 260 images (8.5%). They were as follows: lunato-triquetral carpal coalition (*n* = 7), flexion deformities (*n* = 3), brachydactyly (*n* = 3), dense metaphyseal bands (*n* = 3), polydactyly (*n* = 2), sclerotic bones and acro-osteolysis (*n* = 1), cartilage-capped exostoses of the radius (*n* = 1), old fracture (*n* = 1) and negative ulna variance (*n* = 1).

### Agreement between manual readers and between Manual BA and BoneXpert BA

**FIGURE 3:**
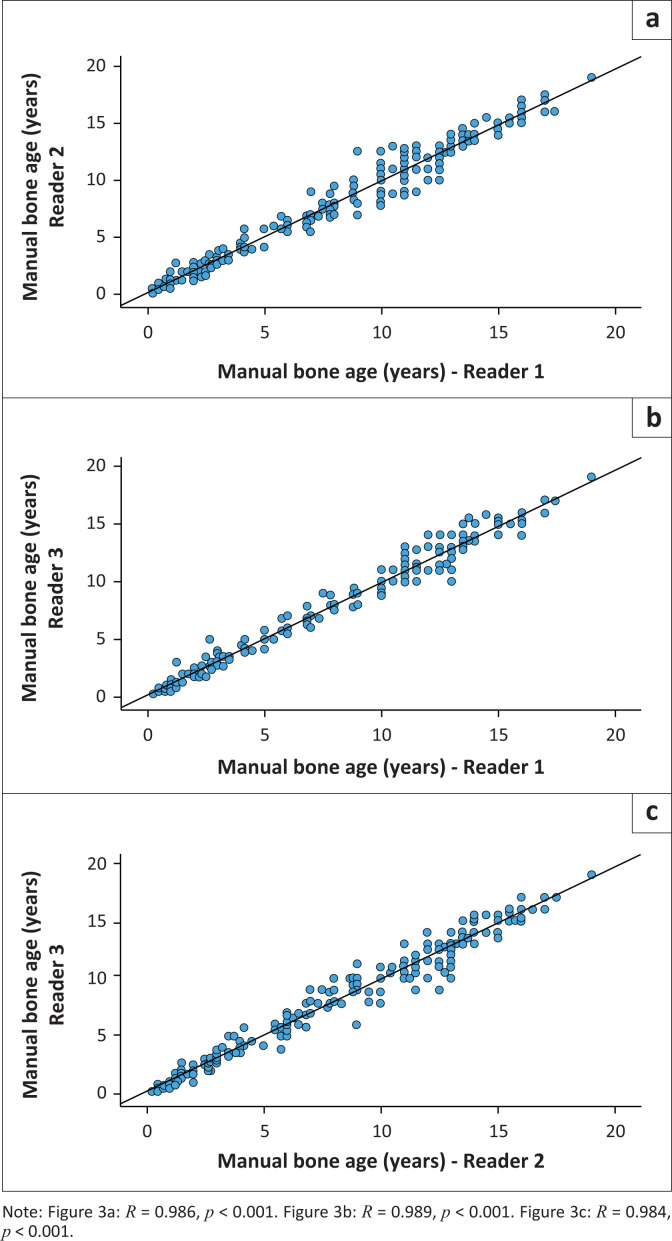
Scatter plots demonstrating the positive correlation between manual bone age Reader 1 and Reader 2, Reader 1 and Reader 3, and Reader 2 and Reader 3.

The ICC between Reader 1 and Reader 2 was 0.986 (95% CI = 0.982–0.989, *p* < 0.001); between Reader 1 and Reader 3 was 0.989 (95% CI = 0.986–0.991, *p* < 0.001); and between Reader 2 and Reader 3 was 0.984 (95% CI = 0.980–0.988, *p* < 0.001) (Figure 3).

**FIGURE 4:**
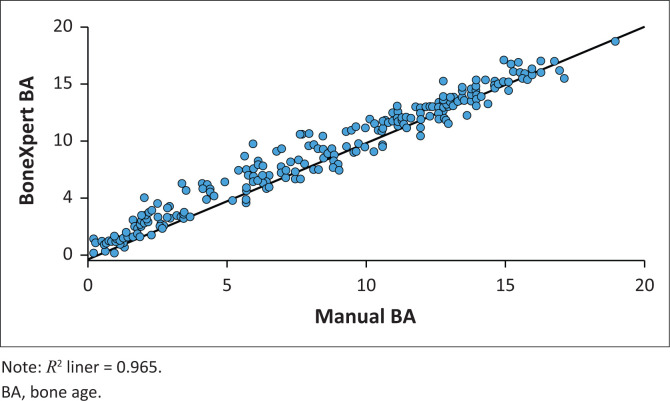
Scatter plot demonstrating the positive correlation between BoneXpert BA and Manual BA.

**FIGURE 5:**
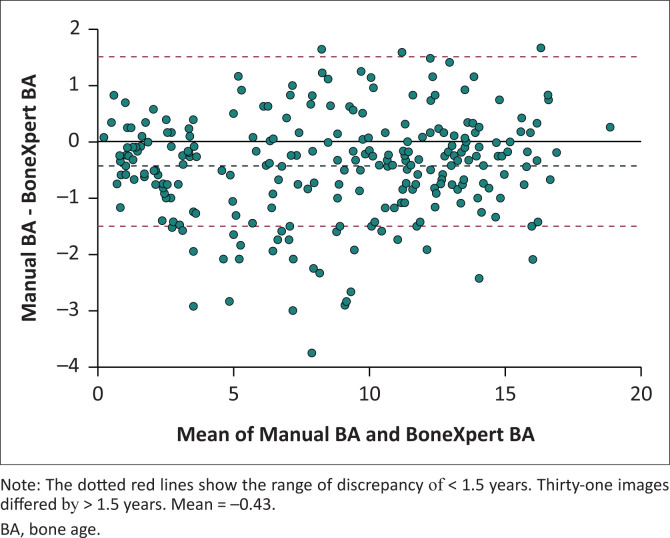
Bland–Altman plot that demonstrates the relationship between the average of the manual bone age readings (Manual BA) and the automated bone age readings (BoneXpert BA).

The ICC between Manual BA and BoneXpert BA was 0.982 (95% CI = 0.977–0.986, *p* < 0.001) (Figure 4). The mean difference between Manual BA and BoneXpert BA was –0.43 years (standard deviation of 0.91 years). The Bland–Altman plot demonstrates the relationship between the Manual BA and BoneXpert BA (Figure 5).

### Analysis of the outliers

Of the 260 images, 31 images had a bone age discrepancy of > 1.5 years between the Manual BA and the BoneXpert BA. These 31 images were re-read by an external reader, producing Reference BA (Table 2). In 23 of 31 images, Reference BA was closer to Manual BA. In eight of 31 images, Reference BA was closer to BoneXpert BA.

**TABLE 2:** Thirty-one images with a discrepancy of > 1.5 years between Manual BA and BoneXpert BA were reread to produce a Reference BA.

Case ID	Gender	Manual BA	BoneXpert BA	Reference BA	Reference BA – Manual BA	Reference BA – BoneXpert BA	Carpal BA	Carpal BA –Manual BA
010	M	9.7	11.2	10.0	0.3	−1.2	9.9	0.2†
012	M	6.0	7.6	7.0	1.0	−0.6‡	80	2.0
028	M	2.6	4.8	2.3	−0.3	−2.5	2.1	−0.5†
030	M	8.0	10.7	7.0	−1.0	−3.7	9.5	1.5
032	M	6.8	9.1	7.0	0.2	−2.1	8.8	2.0
063	M	4.2	6.2	4.0	−0.2	−2.2	4.2	0.0†
101	F	2.3	3.9	2.1	−0.3	−1.8	2.3	0.0†
105	M	8.0	9.6	8.8	0.8	−0.9	9.2	1.2†
123	F	5.7	8.6	4.7	−1.0	−4.0	-	-
127	M	10.2	11.9	11.0	0.8	−0.9	-	-
129	F	12.0	10.4	9.4	−2.6	−1.0‡	-	-
135	M	7.7	10.6	7.5	−0.2	−3.1	7.8	0.1†
171	M	4.2	5.8	4.5	0.3	−1.3	4.5	0.3†
186	M	11.2	13.1	11.0	−0.2	−2.1	-	-
187	M	5.5	7.4	6.7	1.2	−0.8‡	4.6	−0.9†
191	M	3.6	3.7	3.5	−0.1	−0.2	3.7	0.2†
199	F	5.8	7.5	6.8	1.0	−0.8‡	6.5	0.7†
210	M	7.8	10.6	7.0	−0.8	−3.6	12.2	4.4
213	F	7.0	9.3	6.8	−0.2	−2.5	8.2	1.2†
214	M	2.3	3.9	2.0	−0.3	−1.9	1.4	−0.9†
224	M	6.2	7.9	6.0	−0.2	−1.9	5.8	−0.4†
227	F	9.1	7.4	7.5	−1.6	0.1‡	8.5	−0.6†
233	M	4.3	6.2	4.0	−0.3	−2.2	4.2	−0.1†
234	M	6.2	8.3	7.7	1.5	−0.6‡	7.0	0.8†
238	F	17.2	15.5	15.0	−2.2	−0.5‡	-	-
239	M	12.8	15.2	15.0	2.2	−0.2‡	-	-
267	M	6.0	9.8	6.4	0.4	−3.4	6.2	0.2†
269	M	2.1	5.0	1.9	−0.2	−3.1	1.7	−0.4†
271	M	15.0	17.1	15.0	0.0	−2.1	-	-
274	M	8.5	10.4	8.8	0.3	−1.6	8.8	0.3†
305	M	3.4	6.2	4.5	1.1	−1.7	4.1	0.7†

BA, bone age; M, male; F, female; ID, identity.

† , cases where Carpal BA is closer to Manual BA than BoneXpert BA;

‡ , cases where Reference BA are closer to BoneXpert BA than to Manual BA.

The Carpal BA was computed for 24 of the 31 cases. In 20 of 24 cases, the Carpal BA was closer to manual BA than BoneXpert BA and in 4 of 24 cases, the Carpal BA deviated from the manual BA by > 1.5 years.

### Composite bone age

**FIGURE 6:**
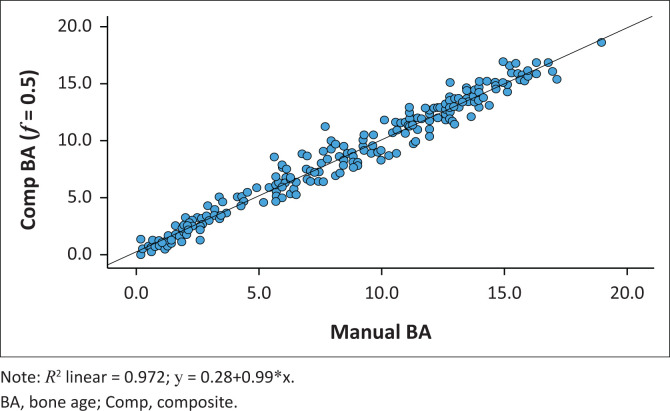
Scatter plot demonstrating the relationship between Manual BA and Comp BA (*f* = 0.5) with 50% weighting on carpal bones and 50% weighting on tubular bones.

**FIGURE 7:**
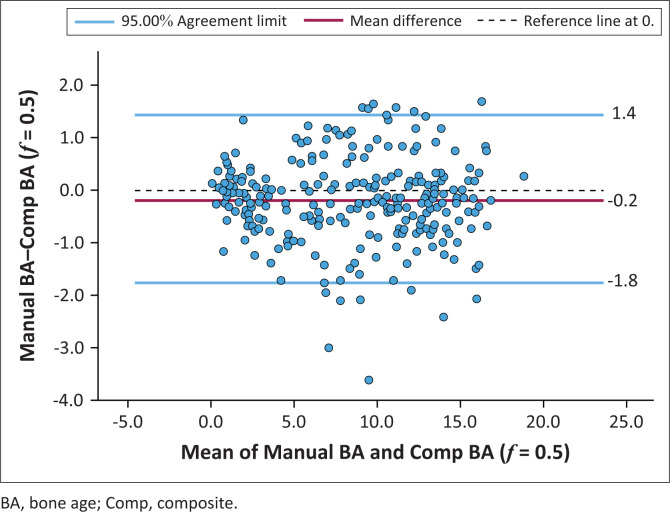
Bland–Altman plot that demonstrates the agreement between average of the manual bone age readings (Manual BA) and composite automated bone age with 50% weighting on carpal bones and 50% weighting on tubular bones (Comp BA [*f* = 0.5]).

A Comp BAwith an f-value of 0.5 achieved the best agreement with Manual BA. The ICC between Comp BA (*f* = 0.5) and Manual BA was 0.993 (95% CI = 0.991–0.994, *p* < 0.001) (Figure 6 and Figure 7). The R^2^ between CompBA (*f* = 0.5) and Manual BA was 0.972, indicating a good fit. The RMSE between Comp BA (*f* = 0.5) and Manual BA was 0.84 years.

## Discussion

BoneXpert software has been validated for some but not all ethnicities.^3,16,17,18,19,20,21,22^ It has also been validated for use in children with short stature,^23^ congenital adrenal hyperplasia,^24^ precocious puberty,^25^ increased body mass index^25^ and diabetes mellitus.^26^ The Johannesburg Birth to Twenty cohort of 363 healthy South African children was used in the development of an African Model for the Adult Height Prediction function of the BoneXpert software in 2021. The current study assessed the bone age assessment function of BoneXpert in a clinical setting in South African children.

The GP atlas was derived from 572 left-hand radiographs of healthy boys and girls in Cleveland, Ohio, United States of America, between 1931 and 1942.^4^ It is well known that the tempo of skeletal maturation differs among populations and ethnicities.^10,27,28,29^ Because of racial diversity, racial mixing and a large immigrant population in South Africa, GP standards for bone age assessment must be used with reservation. Manual bone age assessment using the GP method is subjective^25^ and there is often no perfect match. The GP atlas also categorises bone ages in fixed six-monthly intervals, whereas BoneXpert software assesses bone age as a continuous variable. BoneXpert is therefore a more appropriate measure of the continuous linear process of skeletal maturation and provides a better assessment of response to treatment (e.g., during growth hormone treatment).^14^

This study performed manual and automated bone age assessments on images of typical quality in paediatric radiology practice and in children with paediatric endocrine pathologies. BoneXpert was designed as a clinical tool in paediatric radiology and multiple previous studies have already examined its use in healthy children.^3,16,22,30^ The population of this study was children who attended the paediatric endocrinology clinic and required bone age assessment radiographs for various clinical indications, thus utilising BoneXpert in the clinical setting for which it was intended.

BoneXpert achieved an efficiency of 97.7%, rejecting only a small percentage of images. This is comparable to other studies, where BoneXpert’s efficiency was 98.7%,^23,25^ 99.8%^30^ 99.5%.^17,22^ The reasons for rejections in this study were acceptable with four images of poor quality (foreign objects superimposed on the radiograph and insufficient bones imaged) and two images related to bone pathology (flexion deformities).

Other incidental radiological findings were identified in 8.5% of images. The findings were chronic musculoskeletal pathology (e.g. flexion deformities) and old fractures that would not necessarily influence immediate patient management but are important signs of disease that could have medicolegal implications if missed. A drawback of artificial intelligence tools is that through mathematical algorithms, the software can perform only specific tasks. On the other hand, the human radiologist, through experience and understanding of clinical disease, can holistically analyse images and detect signs of multiple pathologies simultaneously. Only 2 of the 22 images with other radiological findings were rejected by the software. A concern might be that the software accepts too many images and only rejects significantly abnormal images. Subtle abnormalities of bone morphology or texture may be missed because detecting these changes is beyond the scope of the software.^11^ Therefore, although BoneXpert was designed as an AI-replace tool, most radiologists using the software still review the images for signs of disease,^14^ suggesting more of an AI-assist role.

Manual bone age assessment methods are associated with interobserver variability, which renders it less clinically useful. In this study, three manual readers from different specialities read all the images in the data set. There was no significant interobserver variability between the three manual readers, with an ICC of > 0.9, indicating a high positive correlation. Similarly, Bowden et al. reported a significant concordance of 0.98 between two manual readers.^20^ Studies performed by Koc et al.^31^ and Kaplowitz et al.,^32^ also indicated a good correlation between manual readers of different specialities. An important caveat in the current study is that the participating manual readers perform regular bone age assessments and therefore, the interobserver agreement may not be generalisable to all manual readers in daily practice. Furthermore, the images in the data set were read continuously in a batch, so readers became proficient while performing the task repeatedly. The manual readers were also blinded to the subjects’ chronological age and clinical condition, eliminating any bias. However, in a PACS-based environment, it is impossible to blind readers to chronological age. Manual readers are also biased by knowledge of the previous bone age readings and the patient’s clinical history.

This study found a high positive correlation between the manual GP method and BoneXpert method, with an ICC of 0.982. Similarly, a study in a North American population reported a correlation coefficient of 0.98 between the two methods.^20^ In the Brazilian and Chilean populations, the ICC between the two methods was > 0.9 and 0.91–0.93, respectively. (Table 3).^17,18^

**TABLE 3:** Manual bone age assessment versus BoneXpert in other studies in various populations.

Author and year	Region	Clinical condition of study population	Number of radiographs	Chronological age (years)	Manual BA method	Relation between manual BA method and BX
Van Rijn et al. (2009)^3^	Netherlands	Healthy children	531	3.8–20.1	GP method	Manual GP vs BX RMSE: 0.65 years (boys) 0.76 years (girls)
Martin et al. (2009)^23^	Germany	Various diagnoses of short stature	1097	2–17 (boys) 1.5–17 (girls)	GP method	Manual GP vs BX RMSE: 0.7 years (boys) 0.74 years (girls)
Thodberg et al. (2010)^33^	United States	Healthy children	1100	2.5–17 (boys) 2–15 (girls)	GP method	Manual GP vs BX RMSE: 0.61 years
Martin et al. (2010)^16^	Japan	Healthy children and children treated with GH or GnRHa	284	4–21 (boys and girls)	TW Japan system	Manual TW Japan system vs BX s.d.: 0.72 years
Martin et al. (2011)^25^	Germany	Precocious puberty or early puberty	741	0.3–14.8 (boys and girls)	GP method	Manual GP vs BX Mean difference: -0.19 years (s.d.: 0.72 years)
Zhang et al. (2013)^30^	China	Healthy children	6026	2–20 (boys) 2–19 (girls)	TW3 method	Manual TW3 vs BX RMSE: 0.64 years (boys) 0.68 years (girls)
Martin et al. (2013)^24^	Germany	Congenital adrenal hyperplasia	892	0–17 (boys and girls)	GP method	Manual GP vs BX Mean absolute difference: 0.54 years s.d.: 0.4 years
Pose Lepe et al. (2018)^17^	Chile	Children presenting for bone age assessment	1493	< 16 (boys and girls)	GP method	Manual GP vs BX Intraclass correlation: 0.91-0.93 Average difference: 0.19 years
Artioli et al. (2010)^18^	Brazil	Eutrophic, overweight and obese	515	5–17 (boys and girls)	GP method	Manual GP vs BX Intraclass correlation: >0.9 RMSE: +/- 1 year
Alshamrani et al. (2019)^19^	Saudi Arabia	Children presenting for possible bone fracture	420	1–18 (boys and girls)	GP and TW3 method	Manual GP vs BX Average difference: 3 months (boys) 1 month (girls) Manual TW3 vs BX Average difference: 1 month (boys and girls)
Bowden et al. (2022)^20^	United States	Children presenting for bone age assessment	614	1.8–18.7	GP method	Manual GP vs BX Intraclass correlation: 0.98 RMSE: 0.75 years
Oza et al. (2022)^21^	India	Healthy children	920	2–19 (boys and girls)	GP, TW2 and TW3 method	Manual GP vs BX RMSE: 0.39 years Manual TW2 vs BX RMSE: 0.41 years Manual TW3 vs BX RMSE: 0.36 years
Maratova et al. (2023)^22^	Czech Republic	Children presenting for bone age assessment	1285	5–16 (boys) 5–15 (girls)	GP and TW3 method	Manual GP vs BX version 2 RMSE: 0.55 (boys) 0.59 (girls) Manual GP vs BX version 3 0.68 (boys) 0.52 (girls) Manual TW3 vs BX version 2 RMSE: 0.57 (boys) 0.72 (girls) Manual TW3 vs BX version 3 RMSE: 0.51 (boys) 0.49 (girls)
Oza et al. (2024)^26^	India	Type 1 diabetes mellitus	1272	2–17 (boys and girls)	TW3 method	Manual TW3 vs BX Intraclass correlation: 0.983 RMSE: 0.72 years (boys) 0.67 years (girls)

Note: Please see the full reference list of Minty R, Mahomed N, van Wyk NTC, Ranchod AI, Mndebele G, Lockhat ZI. Comparison of bone age assessment using manual Greulich and Pyle method versus automated BoneXpert method in South African children. S Afr J Rad. 2025; 29(1), a3033. https://doi.org/10.4102/sajr.v29i1.3033 for more information.

BA, bone age; GP, Greulich and Pyle; TW, Tanner-Whitehouse; BX, BoneXpert; RMSE, root mean square error; s.d., standard deviation.

The mean difference between the average of the manual readings and automated readings was –0.43 years. Alshamrani et al. observed a mean difference of 3 months in boys and 1 month in girls between the two methods.^19^ A much smaller mean difference of 0.19 years between the two methods was reported by both Martin et al. and Pose Lepe et al.^17,25^

The first validation study performed in healthy Dutch children reported a RMSE of 0.65 and 0.76 years in boys and girls.^3^ Thodberg’s study in healthy American children from four ethnicities showed a similar accuracy of 0.74 years, whereas Oza et al. reported an even higher accuracy of 0.39 years in healthy Indian children.^21,33^ Multiple studies have also compared the manual TW method to BoneXpert, achieving similar results.^19,21,22,26,30^

In 31 images, there was a discrepancy of > 1.5 years between the average of the manual readings and the automated reading. The Bland–Altman analysis found the largest difference between the two methods in patients with a mean chronological age of 9.2 years. This is in keeping with other studies where the greatest differences between the two methods were found at the peri-pubertal age.^18^ These 31 images were re-read by an external reader, producing a Reference BA, which was closer to BoneXpert BA in 8 of 31 images and closer to Manual BA in 23 of 31 images. The discrepancies between manual bone age and automated bone age could be related to the method by which BoneXpert calculates bone age; it uses 21 tubular bones, namely the radius, ulna and short bones (RUS). The carpal bones are evaluated separately to produce a carpal bone age. Carpal BA was computed in 24 of these 31 images and is compared to Manual BA in Table 2. Carpal BA was closer to Manual BA in 20 of 24 images, suggesting that manual readers put more weighting on carpal bones for bone age assessment.

Manual BA readings in this study were lower than BoneXpert BA. Previous studies by Oza et al.^34^ and by Lee et al.,^35^ found that assigning a higher weightage to carpals, results in underestimation of bone age. In an attempt to establish backward compatibility with the Manual BA in the current study, a composite bone with variable weightage on carpal bone age (i.e. 10%, 20%, 30% etc.) was defined and the best agreement between Manual BA and Comp BA was obtained with a 50% carpal bone weighting. Placing 50% weighting on carpals and 50% weighting on tubular bones resulted in an intraclass correlation of 0.993, R^2^ of 0.972 and RMSE of 0.83 years between manual bone age and composite bone age. Similarly, Oza et al. analysed various composite bone ages and found that the best agreement between manual bone age and BoneXpert was obtained by placing 50% weighting on the carpals.^21^ Hence, BoneXpert’s bone age based solely on tubular bones may overestimate bone age in pre-pubertal children. When using BoneXpert as a clinical tool, in cases where the carpal and tubular bone ages are reported separately, a composite bone age should be calculated using a 50% weighting on carpal bones and 50% weighting on tubular bones.

A limitation of this study is that the sample was from a single city. However, the population living in Johannesburg is very diverse, and therefore, a representative sample of the country. Further areas for potential research are examining the Bone Health Index and Adult Height Prediction functions of BoneXpert.

Automated bone age assessment using BoneXpert eliminates interrater variability and reduces the frequency of errors. There is no bias from the chronological age, previous bone age reading and clinical history. This method is time-saving, performing a bone age analysis in less than 15 s. It is advantageous that BoneXpert rejects certain images because, in a busy clinical setting, it red flags images with poor quality or pathology that require further human evaluation.^14^

## Conclusion

On average, the automated BoneXpert method agrees with the manual GP method for bone age assessment in South African children with various paediatric endocrinology diagnoses. 50% weighting on carpal bones and 50% weighting on tubular bones establishes the best agreement between manual bone age and automated bone age. BoneXpert can be used as an AI-assist tool, where the software can accurately and objectively provide bone age readings alongside the radiologist, who can assess images for signs of underlying disease.
